# Hydroxychloroquine Serum Concentration in Coronavirus Disease 2019 (COVID-19) Patients: A Retrospective Study

**DOI:** 10.1080/20961790.2021.1936896

**Published:** 2021-08-19

**Authors:** Saadi Fatima Zohra, Lachgueur Nassima

**Affiliations:** Department of Toxicology, University Hospital, Oran, Algeria; Pharmacy Department, Faculty of Medicine, Oran, Algeria; Faculty of Medicine, Environmental Health Research Laboratory, Oran, Algeria; Department of Toxicology, University Hospital, Oran, Algeria; Pharmacy Department, Faculty of Medicine, Oran, Algeria

**Keywords:** Forensic sciences, concentration, COVID-2019, hydroxychloroquine, HPLC-DAD, plasma

## Abstract

Algeria has adopted a therapeutic protocol using hydroxychloroquine (HCQ) as a first-line treatment for patients with coronavirus disease 2019 (COVID-19). The administration of HCQ must be accompanied by appropriate cardiac and therapeutic pharmacological monitoring to avoid side effects and toxicity. This work is a retrospective descriptive study aiming to estimate the HCQ levels of COVID-19 patients. HCQ concentrations in blood samples from COVID-19 patients were determined using high performance liquid chromatography with diode array detector (HPLC-DAD) after liquid-liquid extraction of plasma. The coefficient of determination (*r*^2^) of this method was 0.9999. The limits of quantification and detection were 0.05 mg/L and 0.02 mg/L, respectively, with coefficients of variance less than 6%. Patient monitoring was performed following recommendations for the therapeutic pharmacological monitoring of HCQ in patients treated for SARS-CoV-2 (COVID-19) infection validated by the coordinated action (AC43) of the National Agency for Research on AIDS and Viral Hepatitis (ANRS) and Therapeutic Pharmacological Monitoring and Personalization of Treatments (STP-PT) group. A total of 267 blood samples from 240 patients were analyzed, 49% for males and 51% for females. More than a third of the patients were 60–80 years old. The majority (68%) of the HCQ concentrations were between 0.1 and 1 mg/L. Additionally, more than 20% were between 0.05 and 0.1 mg/L, while only 7.5% were less than 0.05mg/L and 1.5% were greater than 1 mg/L. In summary, patients with COVID-19 showed a good tolerance to HCQ in the majority of cases.

**KEY POINTS**
- HCQ was proposed to be a promised treatment for COVID-19.
- A reverse phase HPLC-DAD method was validated for HCQ concentration determination in plasma.
- Patients hospitalized for COVID-19 were monitored according to the guideline established by the French National Team AC43 of the ANRS and the STP-PT group.
- Patients showed a very good tolerance to HCQ with no observed cases of toxicity.

## Introduction

Hydroxychloroquine (HCQ) is an analogue of chloroquine (CQ) antimalarial, with fewer side effects, a better safety profile, and fewer drug interactions [2]. *In vitro* studies performed on the virus strains responsible for coronavirus disease 2019 (COVID-19) support the potential antiviral effects of CQ and HCQ [3]. However, evidence regarding their effects remains limited and is a subject of controversy [4].

HCQ can be extremely toxic at high doses. Possible resulting symptoms include a rapid onset of seizures, coma, cardiovascular collapse including QRS widening and QT interval prolongation, and hypokalemia resulting from intracellular changes [5]. Both the therapeutic advantages and toxic effects of this drug are dependent upon the dose and duration of therapy [6]. Dose regimens should aim to reach plasma concentrations greater than 0.1 mg/L and avoid exceeding plasma concentrations of 1 mg/L [1,5]. Because of the importance of this concentration range, pharmacological monitoring of HCQ treatment is essential for a good patients care. The present work is a retrospective study which aims to describe the evolution of HCQ concentration in patients with COVID-19.

## Materials and methods

### Study population and analytical methods

Over 5 months (April–August 2020), patients hospitalized for COVID-19 and treated with 600 mg/day were monitored according to the guidelines established by the French National Team AC43 of the National Agency for Research on AIDS and Viral Hepatitis (ANRS) and the Therapeutic Pharmacological Monitoring and Personalization of Treatments (STP-PT) group [1]. Blood samples were collected in heparinized tubes at different time points after treatment initiation (3rd–10th day) within 1 h before drug administration. Patients with renal and/or hepatic disease were excluded. Plasma was recovered and analyzed the same day or kept in freezer for later analysis.

HCQ concentrations in plasma were determined by high performance liquid chromatography with diode array detector (HPLC-DAD) method, which was fully validated as per European Medicines Agency (EMA) guidelines [7]. CQ was used as the internal standard. Separation of analytes was achieved using a Discovery C18 analytical HPLC column (15 cm × 4.6 mm, 5 µm) at 30 °C using Perkin Elmer diode array detector (DAD) at 260 nm. The mobile phase was composed of acetonitrile-phosphate buffer (85:15, v/v) delivered at flow rate of 0.7 mL/min. Liquid-liquid extraction was performed using a mixture of organic solvents (hexane/ethyl acetate).

### Statistical analyses

Data in this study are expressed as mean ± standard deviation (SD). Microsoft Excel 2010 (Redmond, WA, USA) was used for all data analysis.

### Result interpretation

Results were compared with the residual concentrations included in the update of recommendations for the Therapeutic Pharmacological Monitoring of lopinavir and HCQ in patients treated for SARS-CoV-2 (COVID-19) infection [1].

## Results

The retention times of HCQ and CQ were 3.8 and 4.2 min, respectively. The assay demonstrated a good linear dynamic range of 0.05–2.00 mg/L with a coefficient of determination (*r*^2^) equal to 0.9999. Limits of quantification and detection were 0.05 mg/L and 0.02 mg/L, respectively, with coefficients of variance less than 6%.

In total, 267 blood samples from 240 patients were analyzed, 49% for males (*n* = 118), and 51% for females (*n* = 122). Table 1 displays the distribution of these patients according to age. The average HCQ concentration values (±SD) for the patients on each day from Day 2 to Day 10 except Day 9 are presented in Table 2. Among the 267 samples, 20 were below the lowest limit of quantification (LLOQ; 0.05 mg/L), 61 between 0.05 and 0.1 mg/L, 182 between 0.1 and 1 mg/L and four patient more than 1 mg/L (Table 3). According to days, majority of concentrations of HCQ were between 0.1 and 1 mg/L on all days.

**Table 1:** Age distribution of the study population (*n*=240).

Age (year)	Number of patients (%)
[0–20]	6 (2.5%)
(20–40]	62 (26%)
(40–60]	65 (27%)
(60–80]	80 (33%)
>80	13 (5.5%)
Not given	14 (6%)

**Table 2:** Mean hydroxychloroquine concentrations at different days following treatment initiation in coronavirus disease 2019 (COVID-19) patients (*n*=267).

Day	Mean ± SD (mg/L)	Number
2	0.16 ± 0.08	15
3	0.16 ± 0.13	127
4	0.25 ± 0.20	39
5	0.29 ± 0.13	25
6	0.33 ± 0.25	14
7	0.38 ± 0.24	13
8	0.31 ± 0.27	10
9*	–	–
10	0.55 ± 0.42	4

* Day 9: no collected data.

**Table 3:** Distribution according to therapeutic range fixed for coronavirus disease 2019 (COVID-19) infection treatment (*n* = 267).

	Hydroxychloroquine concentration (mg/L) (Number of samples, %)				
Time after treatment initiation (day)	<LLOQ (0.05)	0.05–0.1	0.1–1.0	1.1–1.2	Number of samples
Day 2	3 (17%)	4 (22%)	11 (61%)	0 (0%)	18
Day 3	15 (11%)	47 (33%)	79 (56%)	0 (0%)	141
Day 4	2 (5%)	6 (15%)	33 (80%)	0 (0%)	41
Day 5	0 (0%)	3 (12%)	20 (80%)	2 (8%)	25
Day 6	0 (0%)	1 (6%)	14 (88%)	1 (6%)	16
Day 7	0 (0%)	0 (0%)	13 (100%)	0 (0%)	13
Day 8	0 (0%)	0 (0%)	9 (100%)	0 (0%)	9
Day 10	0 (0%)	0 (0%)	3 (75%)	1 (25%)	4
Total	20	61	182	4	267

LLOQ: lowest limit of quantification.

## Discussion

There were nearly equal numbers of male and female patients in our study population, with most indivi­duals between 60 and 80 years old. The majority of samples (127/247) were collected on the third day (Table 2). In fact, many patients were released after three days of hospitalization and this by overload. Only unstable cases were kept longer. The average HCQ concentration reached the therapeutic range on the second day ((0.16 ± 0.08) mg/L, *n* = 15), contrary to the findings of a study regarding HCQ in COVID-19 patients conducted by Martin-Blondel et al. [8]. In this case, the mean was still in the infra-therapy range ((0.09 ± 0.01) mg/L, *n* = 3) on the second day following treatment initiation at 600 mg/day while this range was well achieved after the same period with 800 mg/day on the first day then 600 mg/day (*n* = 14). It only on Day 3 that therapy concentration were achieved ((0.10 ± 0.04) mg/L, *n* = 14) with 600 mg/day regimen. However, it is important to note that the number of patient blood samples collected on Day 2 in this study was small (*n* = 3). Perinel et al. [9] conducted a prospective study to evaluate the pharmacokinetic properties of HCQ and reported a 2.7 days (1–4.5 days) mean time to reach the blood therapeutic level. Morrisette et al. [10] reported that a mean peak blood and plasma HCQ concentrations of 0.1296 mg/L after 3.26 h and 0.0503 mg/L after 3.74 h, respectively, were successfully achieved in healthy males who received a single HCQ 200 mg oral dose. This suggests that physiological variations in COVID-19 patients may prolong the time to reach therapeutic concentration.

Interestingly, the average HCQ concentration in our study generally increased each day from (0.16 ± 0.08) mg/L at Day 2 to (0.55 ± 0.42) mg/L at Day 10), except on Day 8 when concentration decreased slightly and Day 9 when no data are available (Table 2).

The distribution according to therapeutic level suggested that most (182/267, 68%) of the measured HCQ concentrations were in therapeutic range, while 61/267 (23%) were below the minimum therapeutic target. Only 4/267 (1.5%) samples were slightly above 1 mg/L without any toxic symptoms. Notably, 20/267 (7.5%) were below the LLOQ (0.05 mg/L). However, this only occurred during the first 4 days of treatment and was likely because those patients did not adhere to the treatment regimen (Table 3). Perinel et al. [9] reported that 6/161 samples (3.7%) in their analysis were below the LLOQ (0.01 mg/L), 61% patients achieved the thera­peutic level, and 15% exceeded the therapeutic level. Our results as well as those reported in other studies show that HCQ reach therapeutic concentration fixed for COVID-19 disease during the first days with no intoxication cases. This makes this molecule at recommended regimen (600 mg/day) save.

## Conclusion

Our retrospective study suggests that COVID-19 patients treated by HCQ following a 600 mg/day regimen were overall mostly sufficiently covered and impregnated by the treatment from the first days after treatment initiation. These patients showed a very good tolerance to this drug with no observed cases of toxicity. We believe that HCQ at this dose is a safe treatment for humans regardless of its effectiveness in treat COVID-19, which remains a subject of great controversy.

## Data Availability

The data supporting the conclusions of this study are available from the corresponding author, Saadi Fatima Zohra, upon reasonable request.

## References

[1] Société Française de Pharmacologie et de Thérapeutique (SFPT). Recommandations et publications. Paris (France): SFPT; 2020. Standard No. ISO 15189 ET/OU 17025. Available from: https://sfpt-fr.org/recommandations-et-publications. French.

[2] Alia E Grant-Kels JM. Does hydroxychloroquine combat COVID-19? A timeline of evidence. J Am Acad Dermatol. 2020;83:e33–e34. 32283236 10.1016/j.jaad.2020.04.031 PMC7151328

[3] Sinha N Balayla G. Hydroxychloroquine and COVID-19. Postgrad Med J. 2020;96:550–555. 32295814 10.1136/postgradmedj-2020-137785 PMC10016880

[4] Takla M Jeevaratnam K. Chloroquine, hydroxychloroquine, and COVID-19: systematic review and narrative synthesis of efficacy and safety. Saudi Pharm J. 2020;28:1760–1776. 33204210 10.1016/j.jsps.2020.11.003 PMC7662033

[5] White NJ Watson JA Hoglund RM , et al. COVID-19 prevention and treatment: a critical analysis of chloroquine and hydroxychloroquine clinical pharmaco­logy. PLoS Med. 2020;17:e1003252. 32881895 10.1371/journal.pmed.1003252 PMC7470382

[6] Abdulaziz N Shah AR McCune WJ , et al. Hydroxychloroquine: balancing the need to maintain therapeutic levels with ocular safety an update. Curr Opin Rheumatol. 2018;30:249–255. 29517495 10.1097/BOR.0000000000000500

[7] An agency of the European Union (Agency EM). Guideline on bioanalytical method validation. London (UK): European Medicines Agency; 2011.

[8] Martin-Blondel G Ruiz S Murris M , et al. Hydroxychloroquine in COVID-19 patients: what still needs to be known about the kinetics. Clin Infect Dis. 2020;71:2962–2964. 32392332 10.1093/cid/ciaa558 PMC7239205

[9] Perinel S Launay M Botelho-Nevers É , et al. Towards optimization of hydroxychloroquine dosing in intensive care unit COVID-19 patients. Clin Infect Dis. 2020;71:2227–2229. 32255489 10.1093/cid/ciaa394 PMC7184449

[10] Morrisette T Lodise TP Scheetz MH , et al. The pharmacokinetic and pharmacodynamic properties of hydroxychloroquine and dose selection for COVID-19: putting the cart before the horse. Infect Dis Ther. 2020;9:561–572. 32740858 10.1007/s40121-020-00325-2 PMC7395206

